# Relational Responsibility: Bringing the Wider Social Environment into the Analysis

**DOI:** 10.1007/s41649-025-00379-9

**Published:** 2025-06-23

**Authors:** Serene Ong, Julian Savulescu

**Affiliations:** 1 Centre for Biomedical Ethics, Yong Loo Lin School of Medicine, National University of Singapore, Singapore; 2 Murdoch Children’s Research Institute, Melbourne, Australia; 3 Oxford Uehiro Centre for Practical Ethics, Faculty of Philosophy, University of Oxford, Oxford, UK

**Keywords:** Special responsibilities, Relationality, Relational responsibility, Filial, Socio-relational

## Abstract

Current conceptions of special responsibilities often adopt a narrow, individualistic lens that fails to consider the broader socio-relational context. In response to this gap, we propose a concept of relational responsibility that emphasises the interconnectedness of individuals and the wider societal context in which they exist. We posit that assigning relational responsibilities should not solely hinge on the voluntary nature of one’s relationships, but rather on the intrinsic value of these connections, as determined by individuals who hold pertinent roles within those relationships or who would be impacted by the definition of value. Our account acknowledges that many responsibilities, especially in caregiving contexts, are not chosen freely, and there should be normative limits to protect individuals from unreasonable burdens. Recognising the role of structural conditions in shaping responsibilities, we argue that collectives with the capacity and resources have an obligation to support individuals by mitigating these burdens and creating just conditions for care. This relational and structural reframing offers a more ethically attuned and practically responsive understanding of responsibility.

## Introduction

Consider Sam, whose elderly parent requires specialised and costly home care after a stroke. The healthcare expenses will be significant, but Sam earns too much to qualify for financial assistance. His relationship with his parent is distant, and he resents the filial responsibility expected of him. His story is a common variation of the demanding filial obligations from caregivers, many of whom must compromise their finances, careers, and personal lives — sometimes for years — to care for ageing parents. Societal expectations dictate that we owe filial responsibilities to our parents in ways we do not to others. In Singapore, where an estimated 210,000 caregivers provide support (Ministry of Health 2010), cultural norms strongly emphasise family members’ duty to care for the elderly (Chia and Lee 2022; Rozario and Rosetti 2012). Sam’s case highlights a persistent ethical question: Despite his reluctance, does Sam still have a moral obligation to care for his parent?

While there are general moral responsibilities that we hold to all moral agents (Brown et al. 2019; Hedlund 2012), special responsibilities arise from particular relationships we hold with defined groups of people such as family (Jeske 2021; Murphy 2011; Nagel 1986). Filial responsibilities are a type of special responsibilities. These responsibilities often compel us to act based on the relational context, even when those actions may conflict with our personal preferences. However, the focus here is not on responsibilities as individual choices, but as moral obligations that arise from relational ties. These obligations can require individuals to set aside personal desires or interests to fulfil the needs of others within these relationships.

Some scholars have argued that special responsibilities are personal and supererogatory (Nunner-Winkler 1984) and thus do not constitute fully moral obligations. For example, Lawrence Kohlberg et al. (1983) contended that responsibilities grounded in personal relationships may be considered extra-moral, as they exceed the basic ethical expectations owed to others in general society. If we take a relational perspective, however, special responsibilities are in fact morally significant because they recognise the interdependence of individuals and the importance of contextualised ethical duties within relationships (Held 2005). This view challenges traditional notions of autonomy and suggests that moral obligations are not solely grounded in universal principles, but also in the lived realities of relational interconnection. It offers a more nuanced understanding of moral duties by including the moral weight of our connections with others. Much has already been discussed in the literature on the justification and nature of these special responsibilities (Jeske 2021; Pettit and Goodin 1986), and we agree summarily that agents do hold special responsibilities towards specified others.

A common scheme to describe responsibility (Duff 2009) is commonly laid out as:

*A* is responsible to *B* for *X*,(1).

where *A* and *B* are agents, and *X* is some desired state of affairs.

While this formulation is analytically useful, it presumes a free-floating individual-centric formulation of responsibility. Although some accounts note how social determinants such as material circumstances, education, and occupation can affect an agent’s behaviour significantly to the extent that they may not be fully fit to be held responsible for their health behaviour (Brown 2013; Pettit 2001), they often fail to engage with the larger socio-relational structures that link A and B, including the presence of other relevant agents, cultural norms, or institutional systems that mediate their relationship.

Dominant approaches tend to treat responsibilities as static, dyadic obligations between two parties without considering the wider network of relationships, institutional structures, and socio-cultural norms that shape and mediate these obligations. As a result, these debates place disproportionate emphasis on the moral duties of individual agents while neglecting systemic and institutional factors that contribute to inequities within care, such as underfunding or lack of support for caregivers. In addition, special responsibilities that prioritise certain relationships may do so at the expense of others. Sam would likely have multiple relational obligations — placing filial duties on Sam without considering his broader relational context risks neglecting other relationships and obligations, leading to moral distress and burnout.

This paper advances the concept of relational responsibility, which reframes special obligations not as free-floating duties between isolated agents but as obligations shaped and constrained by social structures, institutional norms, and a multiplicity of interwoven relationships. Our argument is that relational responsibility offers a more context-sensitive and normatively grounded account of how caregiving obligations arise and how they ought to be distributed.

We make three key contributions. First, we extend existing care-based and relational ethics by developing a framework that explicitly integrates structural critique into the moral evaluation of caregiving responsibilities. Second, we reframe moral agency by showing that responsibility arises not only from direct relationships (e.g. parent–child) but also from indirect relational entanglements, including with institutions, norms, and communities. Third, we expand the discourse on moral responsibility by showing how involuntary caregiving roles often reflect broader patterns of structural injustice that should be redistributed across the collective rather than left to individuals alone.

Our concept of ‘relational responsibility’ widens the analytical focus to ask: what do special responsibilities mean for agents who are embedded within a complex web of social structures and relationships? How does the myriad of relationships affect and shape these special responsibilities?

*Relational responsibility*: The unique obligation an agent, *A*, has towards a specified agent, *B*, that arises from the specific relationship *A* has with *B* as well as the broader socio-relational environment *A* and *B* are located within.

*Socio-relational environment*: The relational environment that comprises relationships with other relevant agents, and the structural environment that comprises socio-cultural norms, historical circumstances, political landscapes, legal standards, and financial structures.

Throughout this paper, we use the term *responsibility* in a broad sense to refer to a moral orientation or accountability towards others that may include, but is not limited to, *obligations*. While obligations often imply specific, action-guiding duties — such as those codified in professional codes or legal frameworks — responsibilities can also involve informal, relational, or role-based expectations that are shaped by social and structural contexts (Young 2011; Tronto 1993). In caregiving and healthcare, for example, one may feel a responsibility to care for others that is not easily reducible to a discrete obligation. Our account of relational responsibility is intentionally framed to capture this broader, more flexible conception.The scope of this paper is limited to significant caregiving responsibilities in the healthcare context, particularly those that are long-term and burdensome. While it is true that we can hold special healthcare-based responsibilities to friends and communities, pertinent in the wake of COVID-19 (Ryan 2023), our focus is on familial care, where these burdens are most deeply entrenched and institutionally neglected.

As our account overlaps with common understandings of special responsibility, we use the term ‘special responsibility’ when we refer to common understandings in the literature, and ‘relational responsibility’ to distinguish our account. We start by exploring the normative basis for relational responsibilities, including the common objection that not all relationships are freely chosen. We argue that some involuntariness is inevitable in ascribing relational responsibility; nonetheless, there ought to be normative bounds on such involuntary responsibilities. We then explain why the socio-relational environment should be given more explicit attention. On this basis, we argue that relational responsibility is inherently polyadic and context-sensitive, extending across networks of agents and shaped by structural conditions. In the final sections, we discuss the implications of this view. Specifically we consider how collective responsibility, particularly in institutional and policy settings, can mitigate the burden of involuntary relational responsibilities, and how structural factors can and should also be put to work to create reasonable solutions or alternatives for individuals.

## Relational Responsibility

As relational responsibility builds upon special responsibilities, we begin by considering the nature and the justification for special responsibilities. Moral responsibility is highly interpersonal. Peter Strawson (2008) highlighted how our reactive attitudes, like resentment and gratitude, shape acts of blame, praise, punishment, and reward. Thomas Scanlon (2008) argued that blame arises within relationships, explaining why people react differently to wrongdoing based on how it affects them. Blame, then, is not just a reaction but a morally appropriate response in certain contexts. Specific interpersonal relationships also grant us the ‘moral authority’ to hold another responsible (Radzik 2011) — for example, parents may discipline their children, but disciplining another child requires granted authority, such as that of a teacher.

### Extending from Relational and Care-Based Ethics

We draw on feminist conceptions of relationality (Koggel et al. 2022), which hold that an individual cannot be abstracted from their environment, and their decisions, actions, and values must be analysed within the context of this network. This approach contrasts with more traditional conceptions of persons as independent and self-interested individuals. Relationality is thus a ‘shaping sensibility’, a prism that informs other values and principles such as autonomy, justice, and responsibility (Jennings and Dawson 2015).

While our account of relational responsibility draws on several well-established relational moral theories, it offers a distinct contribution by bringing together structural critique and moral obligation within the specific context of caregiving. Previous influential work by feminists, communitarians, and virtue ethicists has emphasised relational embeddedness, but few have examined how moral responsibilities in care settings are shaped and constrained by broader socio-relational and institutional forces, and how these generate distinct normative pressures that go beyond mere relational interdependence.

Influential work by care ethicists including Joan Tronto (1993) and Eva Kittay (2001) has emphasised dependency and relational care. Tronto, for instance, wrote that care is not merely a private act but a political and institutional concern. We build on her work by shifting attention from the act of caring to the *attribution* of responsibility for care: who gets assigned moral responsibility, under what conditions, and with what support. We argue that even when care is ethically important, the way responsibilities are assigned can be unjust — particularly when agents are involuntarily burdened. Alasdair MacIntyre’s (1999) critique of liberal individualism highlighted the dependency and social embeddedness of moral agents and focused on the cultivation of virtues within communities. In this paper, our concern is with the normative burden placed upon individuals by socially and culturally generated roles and expectations, especially when those roles are not voluntarily assumed.

Iris Young (2011) argued that we are responsible for structural injustices because we are all socially connected participants in the systems that produce them. Our account builds on their insight by bringing it into the domain of interpersonal caregiving, where structural injustices such as policy gaps, institutional neglect, and normative expectations can produce private moral burdens. While their account focuses on structural political and economic injustice, we focus on moral responsibility within care relationships, where familial obligations can reflect systemic imbalances and reproduce structural harm when left unsupported.

Onora O’Neill (2000) similarly challenged universalist moral theories that overlook real-world constraints. She argued for attention to conditions of agency, power asymmetries, and institutional feasibility. Our account develops this point further by arguing that justifications for special obligations such as filial care must account for how they are distributed and whether they are supported or imposed unfairly.

These past works have pushed back on individualist ethics and help contextualise moral responsibility within relational and structural frames. Our account advances this discussion by offering a normatively grounded and structurally aware framework for evaluating how caregiving responsibilities arise, are justified, and should be distributed or supported.

### Agent Relativity

We argue that responsibility is inherently relational and agent-relative. The nature and extent of this responsibility depends on the relationship between the responsible agent *A* and the recipient *B*. Moral judgements are often discussed as agent neutral (Nagel 1986), but in reality, most people make moral decisions that are strongly influenced by the identity of the other and the relationship they have with the other (Lee and Holyoak 2020). Indeed, there are a variety of competing deontological moral theories that are agent-relative (Schuessler 2020), and we recognise moral duties towards family differ from those owed to strangers (Weijer 2001).

The specificity of the A–B relationship thus generates non-fungible responsibilities: the value or significance *A* attaches to their relationship with *B* determines the nature and extent of special responsibilities *A* perceives. Consider the following:

- If Alice and Bob have a genetic relationship, and Alice discovers she has a hereditary condition, she might hold a responsibility to inform Bob so that he can get tested.
- If Alice and Bob do not have a genetic relationship, but they are happily married, Alice might still have a responsibility to inform Bob because of their emotional intimacy.
- If Alice and Bob are divorced and have no children, Alice is unlikely to have a responsibility to inform Bob.
- If Alice and Bob have a child who stays with Bob, Alice might have a responsibility to inform Bob because of the implications for their child.
- If Bob is a distant relative, Alice’s perceived responsibility diminishes accordingly.

Throughout these variations, we can see that Alice’s responsibility to Bob changes depending on their relationship.

Indeed, we can see that *A* can hold multiple responsibilities towards different agents, and the nature and extent of these responsibilities changes depending on the different relationships. Say *A* has COVID-19. *A*’s responsibility to see to their own well-being would include self-care actions such as getting plenty of sleep and water, and avoiding alcohol. *A* would also hold a responsibility to individuals whom they have close contact with to reduce chances of infecting them. This responsibility would consist of actions such as isolation or masking up, and these individuals may include family members whom they are living with, neighbours, and the healthcare professionals they encounter at the clinic.

### The Socio-relational Environment

A relational view of the embedded individual stresses the ways in which cultural and social context affect individuals (Mackenzie and Stoljar 2000). A bioethical analysis of the individual must therefore include the relationships individuals have with their socio-relational environment. For example, how does Sam’s filial responsibility change if he has siblings? Does this filial responsibility change if Sam is living in Singapore, where there is a strong expectation of filial responsibilities, as compared to Sweden, where the government is generally expected to provide for the healthcare needs of their elderly citizens?

Taking a relational interpretation of responsibility demands that we consider the *depth* and the *breadth* of the structural context. By depth, we refer to environmental elements such as cultural norms, institutional policies, and legal frameworks that can enable or constrain moral agency. These elements are not moral actors; nonetheless, they can have a direct and causal influence on our capacity to undertake and discharge moral responsibilities. Breadth refers to the multiplicity of these influences, which includes non-human institutions such as governments and economic systems, and the interconnectedness of various relationships. We also understand ‘relationships’ to also include relationships to structural factors; for instance, an individual’s tie to their community, country, financial structures, and legal standards. These can be distinct from an individual’s relationship to other persons in the community and country. For instance, independently from interrelationships with people in their country, one can experience a sense of connection to their country.

Michael Sandel (1998), for instance, argued that an individual is often burdened by inherent commitments stemming from entanglements within the community, rendering them incapable of truly free choice. Such causal conceptions of environmental influences acknowledge the impact of social relationships and social, cultural, and historical circumstances on agents. They view the effects of relational factors as external to the individual and presume that it is feasible to separate the individual from their surroundings. Ideally, the individual can freely express their authentic choice when accepting responsibilities.

We question such presumptions, and posit that it is simply not possible or desirable to detach the individual from their environmental entanglements. An individual’s ascription of responsibility depends very much on their socio-cultural background (Pharr et al. 2014), socio-political policies (Tsutsui et al. 2014), and their perception of their relationship with others. Environmental factors are not merely external to an individual, but can be constitutively defining; i.e. they can fundamentally shape the individual’s view. For example, a study found declines in perceived filial obligations by family caregivers after Japan implemented a mandatory social long-term care insurance system in 2000, which entitles every person over 65 years to caregiving-related services ranging from home help to rehabilitation (Tsutsui et al. 2014). Notably, the shift in a long-held social norm occurred within two years of the policy change and illustrates how public policy can reconfigure moral expectations. Relationality asks how these constitutive commitments and influences shape our views of perceived relational responsibilities, and how others can affect these relational responsibilities.

Ethical analysis, typically concerned with issues of social justice, tends to focus on the identification and removal of negative structures that constrict autonomy and distribution of options available. However, we would not have been able to develop our sense of self without the support of positive social structures. Rather than a free-floating notion of autonomy, relational interpretations reframes autonomy in terms of ‘the forms of human interactions in which it will develop and flourish’ (Nedelsky 2015, 21). While some social structures and relationships are preferred, it must be remembered that all structures and relationships have contributed in some way to our so-called authentic set of values and desires. Reflecting upon these experiences and social influences on our identities will help us distinguish the supportive relationships that are necessary for the healthy development of personhood from oppressive ones. Furthermore, value can be found in the diverse array of relationships we hold. Indeed, not all negative relationships and experiences are terrible — philosophical traditions such as Stoicism and Buddhism view hardship as an opportunity for personal growth and character development rather than something to be avoided or lamented (Irwin 2009).

### Beyond Methodological Individualism

Rebecca Brown and Julian Savulescu (2019) have highlighted that individual responsibility is often shaped by and shared with other agents, and put forth an account of responsibility that paid explicit attention to this dyadic aspect of responsibility. Expanding the scope of our analysis to include more agents and elements often leads to complex and possibly intractable negotiations involving multiple stakeholders with conflicting interests. Reaching consensus or finding mutually acceptable solutions becomes more difficult, potentially resulting in deadlocked or prolonged disagreements.

Nonetheless, disregarding such complexities leads to an impoverished understanding of responsibility. We need to move away from a methodological individualistic approach, whereby social phenomena are analysed and explained in terms of individuals and their properties.

Recall the case of Alice and Bob. They are situated in a social network of others, and these connections can be interdependent. Say Alice has a hereditary condition, and that she has a spouse (Bob) and children. While the relational responsibility Alice has to every other individual can be analysed separately, these responsibilities are not independent but are often co-determinant in affecting other responsibilities. Say Alice holds a responsibility to disclose her condition to her family members so as to ensure their well-being. Alice informs Bob first. What was not initially considered in scheme (1) was that Bob shares a caregiving relationship with Alice. Say Bob offers to inform their children instead; Alice’s responsibility to her children is therefore discharged when she informs Bob. Indeed, when Alice informs Bob, a new responsibility is laid upon Bob to ensure that their children are informed if Alice does not do so.

### Justification for Relational Responsibilities

While there are a number of justifications for special responsibilities (Goodin 1986), we put forth two justifications for relational responsibilities: (i) the value of the relationship and (ii) special responsibilities of care. An important implication of the first justification is the admittance of involuntary relational responsibilities. We argue that relational responsibilities can exist even if people do not want them, because these responsibilities are not grounded in one person’s valuing of the relationship but the value of the relationship itself.

First, relational responsibilities can be justified based on the inherent value of the relationship. Relationships, such as those between family, romantic partners, friends, and colleagues, are often considered valuable because they contribute to individuals’ well-being, personal growth, and sense of belonging (Thomas et al. 2017; Wissing et al. 2021). Relationships are also valued for fostering social cohesion (Friedkin 2004). Relationality argues that recognising these relationships, and derivatively, fulfilling obligations arising from relationships, is essential to promote a flourishing community.

Interestingly, favouritism to certain groups is regarded under certain circumstances, not as prejudice against others or a factual phenomenon, but as morally desired behaviour. We tend to hold special responsibilities above and beyond general moral responsibilities. For instance, we would hold a parent’s responsibility to their children over the parent’s responsibility to any other child whom they do not know. The empirical literature has shown that individuals do ascribe responsibilities arising from familial ties (Dheensa et al. 2016; Lindemann 2019; Miller 2001), which are not based on normative arguments but on the subjective valuations of individuals who hold this value. Jon Leefmann et al. (2017) noted that people can feel obligations to act because of their social roles and identities, even if they cannot always justify this sense of responsibility.

We hold that relational responsibilities arise from the relationship itself. It is the significance we attach to a certain relationship with another that determines the nature and extent of the obligation we perceive. We are often enriched positively by social relationships, and we readily ascribe obligations in response. However, what if we do not value a particular relationship; do obligations arising from that relationship still exist? Under certain circumstances, yes.

Suppose Sam feels completely indifferent towards his parent and does not perceive any filial responsibility. His parent neither abused nor neglected him in childhood; perhaps Sam’s apathy stems from clinical depression or simply his personality. Despite this, he nonetheless holds a relational responsibility to his parent. This responsibility can be justified on their biological link (Callahan 1985), the deep history they share (Dixon 1995), and the parent–child relationship (Nelson 2002) — all of which underscore the inherent value of their bond. Beyond personal connections, social norms, national policies, and legal statutes also affirm the significance of filial responsibility. For instance, Singapore’s Maintenance of Parents Act entitles Singapore residents over 60 years of age to claim maintenance from their children. Such policies reflect prior social priorities that reinforce filial responsibilities in adulthood.

The second justification is care-based: to have a relationship is to have special responsibilities of care towards another person. Robert Goodin (1986) argued that special responsibilities arise from the vulnerabilities of those to whom they are owed and are grounded in values such as compassion, care, and reciprocity. As parents have taken care of children, so adult children have reciprocal duties of care and gratitude towards their ageing parents (Wicclair 1990).

Beyond obligation, relationships also create expectations. *B* may rely on *A* to fulfil a certain responsibility, making *A*’s role essential to *B*’s well-being. The agent-relativistic nature of special responsibilities means that a particular special responsibility owed to *B* is held only by *A*. Societal norms can reinforce these expectations — if society assumes that Sam will care for his parent, this may limit alternative care options, deepening the parent’s vulnerability. We acknowledge this challenge in our account of relational responsibility and address ways to mitigate it by appealing to notions of collective responsibility and reasonable alternatives.

Not all relational responsibilities are moral in nature, but dependency generates some moral implications even in non-moral relational responsibilities because others are relying on the agent to carry out certain responsibilities. For instance, *A* would reasonably hold greater responsibility to their child than to another adult family member because of their child’s greater vulnerability. Moreover, if *A* was a single parent, their responsibility to their children would arguably be greater than if they had a partner to share the parental responsibility.

However, we recognise that relationships can be both empowering and oppressive, and that in some cases, expecting relational responsibilities may be unjust. In the next section, we explore involuntary relational responsibilities and the challenges posed by repressive relationships, addressing common objections to special responsibilities in greater depth.

### The Voluntarist and Oppressor Objections

One common objection to special responsibilities is that not all relationships are freely chosen; consequently, the associated responsibilities are not voluntarily undertaken. Additionally, even freely chosen relationships can sometimes be oppressive. We argue that while relational responsibilities stemming from involuntary relationships can be justified, there must be normative constraints to limit their extent. Rather than grounding relational responsibilities purely in voluntariness, we propose that they should be based on the value of the relationship. This value should be determined by those directly involved in the relationship or those affected by how value is defined. That said, even when a responsibility is ascribed, there are limits to what one person can be expected to do for another. The extent of obligation should be constrained by both the voluntariness of the duty and the capacity of the person bearing it.

Voluntarists accept special responsibilities, but argue that *genuine* special responsibilities must be based on consent or some voluntary act that grounds that responsibility (Scheffler 2002). Call these voluntary special responsibilities. Merely being in a relationship to another does not generate a special responsibility to the other. Voluntarists argue it is unfair to ascribe obligations that are not freely undertaken, or involuntary special responsibilities. The moral force of special responsibilities in this case derives from the fact that they were voluntarily undertaken (Goodin 1986). However, if our relationships to certain people can generate responsibilities independently of our choices, then we may be locked into special responsibilities that we did not choose. This appears troubling.

There is obvious appeal in the ability to voluntarily accept obligations. However, this is often not the case. Relational theory holds that individuals are always socially situated and socially formed. We exist within a social world where some relationships are chosen, while others are not. Consequently, we are bound by obligations beyond our control. We do not choose the family we are born into, or the country we are born in — we enter the world with a predefined structure of obligations and liabilities, in virtue of our particular place in the world. Voluntarists may object to involuntary obligations, yet they remain an undeniable reality of our lives.

Moreover, there is uncertainty in future events. The relationships we voluntarily enter are likely to change with time. Should we continue to be bound to responsibilities arising from these voluntary relationships? To assert that the source of special responsibilities must lie in voluntary acceptance of the responsibilities does not capture the nuances of why people take on special responsibilities, even those that are burdensome. Whether relationships arose voluntarily or involuntarily, we hold onto relationships that we value. In our view, acceptance of relational responsibilities should not be split on lines of voluntariness but value. What follows are relational responsibilities grounded on relationships that are of value. Depending on the nature of these relationships, the people we owe responsibilities towards reciprocate the responsibilities towards me. The burden of these responsibilities is shared if these responsibilities even are burdensome in the first place.

However, using the criteria of value can be vague and subjective: who determines the value of a particular relationship? We suggest that the value of a relationship should be determined by agents who play relevant roles in that relationship, or who would be affected by its definition. For example, say Sam and his sister Anne share a filial responsibility to their parent. Anne has a legitimate interest in asserting that Sam also upholds that responsibility, as his refusal would place the entire burden on her. Indeed, an advantage of having multiple perspectives in determining a relationship’s value is that it acts as a safeguard against biased or distorted judgements. This safeguard is particularly important in cases where individuals may hold onto relationships they fear leaving. For instance, an abused spouse may claim to value their marriage while being unable or unwilling to recognise its oppressive nature. In such cases, relevant agents — such as family, friends, or professionals like social workers — play an important role in assessing whether the relationship truly holds value or is shaped by coercion and harm.

Using the criteria of value rather than voluntariness also takes into account that relationships are dynamic and change with time. People may voluntarily enter relationships, but friends drift apart and couples split up. People may also involuntarily be saddled with relationships they did not choose, but come to value those relationships with time.

The second concern is that some relational responsibilities may appear coerced or involuntary. However, we argue that involuntary responsibilities are not inherently problematic; what matters is distinguishing between enriching and oppressive obligations (Jennings 2016). Regardless of an individual’s obligations, their rights and welfare remain morally significant, and there must be normative limits to the claims others can make upon them (Mackenzie 2014). Our concept of relational responsibilities is grounded in relational individualism, whereby obligations can and ought to be mitigated by other moral values that limit the strength and extent of these responsibilities.

For instance, familial responsibility, often couched as a cultural practice or historical tradition, is susceptible to so-called traditionalist arguments on special responsibilities (Goodin 1986). Empirical studies consistently show that daughters bear a disproportionate burden of informal and long-term caregiving for the elderly (Bott et al. 2017; Dwyer and Coward 1991). Differential filial expectations can take the choice from the caregivers. In Norway, elderly women receive 34% more formal care when they have a son rather than a daughter, as care managers assume daughters will provide more informal support (Jakobsson et al. 2016). It becomes a self-fulfilling expectation as daughters have to compensate for gaps in formal care. Globally, females perform an estimated 75% of unpaid care and domestic work (Moreira da Silva 2019). While some unpaid caregiving is expected, that women shoulder the bulk of unpaid care under the claim of conventional roles or the ‘common good’ (Kittay 2001) is unreasonable.

Acknowledging involuntary special responsibilities does not mean that voluntariness is irrelevant or that responsibilities are unlimited; rather, voluntariness helps determine their strength and extent. In egalitarian relationships, such as those between spouses or friends, both parties freely enter the relationship and stand on equal footing. If *A* fails to fulfil their responsibility to *B*, it would be unreasonable for *A* to expect *B* to uphold theirs. These responsibilities are reciprocal and conditional, arising from mutual choice (McKenna and Coates 2023). Moreover, even for voluntary obligations, there are limits to how much we can expect one person to do for another.

Consider next the relationship between a parent and child. While a parent chooses freely to have a child (assuming ideal conditions), the child did not choose to be born. Is it fair to ascribe filial obligations on the child? At one end, Jane English (1992) answered that while the child may want to provide for their parent, the child does not *owe* their parent. At the other end, Christina Sommers (1986) argued for strong filial obligations grounded on an account of promise-keeping. On her view, parents entered into agreements with their children, whether implicit or explicit, to provide care to the children as they grow up. In return, children are expected to reciprocate by fulfilling filial obligations to their parents later in life. Her promise-keeping account is grounded on fairness and reciprocity. Simon Keller (2006), however, pointed out that filial obligations cannot be understood as debts or reciprocal duties. Instead, he put forth the special goods theory, which holds that the parent–child relationship provides special goods that are generally not easily obtained from other relationships. Consequently, children owe filial obligations because their parents cannot get these special goods elsewhere. Importantly, Keller noted the limits of these obligations: if parents fail to fulfil their own responsibilities, their children’s obligations may be weakened or even dissolved.

One challenge in admitting involuntary special responsibilities is that ascription of special responsibilities is based on a subjective valuation of relationships (Richards 2010), a sensitivity English had acknowledged. She framed the relationship between parent and child as one of friendship and asserted that the associated responsibilities end when the friendship ends. Nicholas Dixon (1995), however, countered that deep relationships retain residual duties even after ending.

Let us return to the issue of normative individualism. Parents often make considerable sacrifices for their children that they do not make for others. If the parent has spent an exorbitant amount of money on the child as the child is growing up, is it fair to expect the child to reciprocate with an equivalent amount in return? Reciprocation expectations must consider factors like relationship history and the child’s circumstances. Generally, if the parent has taken care of the child in their youth, it would be reasonable to expect the child to provide for the parent in old age, but not in excess. Singapore’s Maintenance of Parents Act exemplifies this balance by granting elderly parents unable to maintain themselves a legal claim to basic needs, rather than to the preservation of their previous standard of living. This establishes a base level of reciprocal responsibility constrained by individual capacity.

If we hold that people can ascribe responsibilities arising from relationships they value, then people can also reject responsibilities from relationships that are not of value. That is fair, but the interests of whom the responsibility is owed should also be considered. One issue is that relationships may be asymmetrical. Studies have shown that people in the same relationship may not perceive the same emotional proximity (Cowley 2016). Moral conflict can arise if *A* subjectively denies a relationship with *B* and rejects the linked special responsibility, but *B* expects certain obligations. We would ask why *B* expects a certain obligation, and is it reasonable? Admitting involuntary responsibilities protects *B*, while relational individualism protects *A*. These various considerations are applied to shape the relational responsibility, if any, ascribed onto *A*.

## Implications of Relational Responsibility

Our account of relational responsibility has two key implications: (i) it inherently includes collective responsibility, and (ii) it opens the discussion to how collective responsibility can mitigate involuntary responsibilities. Beyond examining how socio-relational factors shape responsibility, we ask what role these factors can play in supporting individuals. We argue that collectives with the capacity and means have a moral duty to help alleviate the burden of relational responsibilities on individuals.

### Collective Responsibility

While some have critiqued the notion of collective responsibility (Lewis 1948; Narveson 2002), we hold that it is possible to ascribe responsibility to collectives (Cooper 1968). More importantly, collectives can behave in ways distinct from their constituent parts and do work that cannot be done by individuals. Examples of such collectives include sports teams, research teams, and legislative bodies. Peter French (1984) has described formal organisations as moral actors, as these organisations have internal decision structures that make collective decisions and actions possible. The decision structure also allows organisations to respond to backward-looking liability and forward-looking ascription of responsibilities. In such contexts, the collective is more than just an aggregation of individuals, and it would not make sense to analyse the actions of the collective as simply the collective actions of individuals.

Recall Scheme (1). Say *B* has cancer, and *X* is ‘cancer treatment’. An individual on their own may not have the resources to carry out *X*; it will take a team of healthcare providers, together with institutional support from a hospital, to provide effective care for *B*. Consequently, we can think of *A* as ‘the healthcare team plus the healthcare system’. Of course, we can break *X* down into individual-sized chunks *X1*, *X2*, and so on, where *X1* might be ‘taking the patient’s history’, *X2* is ‘carrying out an MRI scan’, and so on. However, this methodological individualism is unable to articulate the norms, structures, and power present within the wider collective of individuals. Moreover, analysing in terms of individual actions does not consider the interdependence of these actions. For example, the surgical team must work in concert to remove the tumour from *B*. For each member to carry out their responsibility effectively, they must be aware of what everyone else in the operating room is doing and act in response to the actions of others.

As shown by the above example, responsibilities can be polyadic. Adopting a relational approach enables us to broaden our perspective and discover the overarching structure of how responsibilities link individuals together. It is crucial to uncover potential larger structures, as collective identity and behaviours may differ from those of individuals. For example, it has been well documented that the behaviour of collectives such as mobs can be strikingly different from the behaviours of individuals on their own (Lama 2021). As such, an analysis of responsibilities that focuses on the individual level when those responsibilities simultaneously involve a group of individuals risks missing essential dynamics when responsibilities are distributed across collectives.

### Many Hands and Reasonable Alternatives

Accepting that relational responsibilities may not always be freely chosen presents a challenge. Even with normative constraints, the burden on individuals can remain substantial, complicating efforts in translating normative ideals into practice. We therefore ask what work the structural environment can do in supporting individuals who bear relational responsibilities, especially involuntary responsibilities.

Collectives such as families can help distribute responsibilities, reducing the burden on individuals. Each member bears partial responsibility, allowing others to step in when some are unable to fulfil their duties. However, this also means that if some members neglect their responsibilities, the remaining members must compensate.^1^

External structural features can also shape and redistribute responsibilities. In the context of responsibility for climate change, Nihlén Fahlquist (2019) argued that if large collectives such as governments and corporations can provide reasonable alternatives at a reasonable cost, they should do so. This reasoning applies to the healthcare setting as well. Consider a patient who tested positive for a hereditary condition and is advised to inform her family members of their genetic risk. While current approaches to familial disclosure place that responsibility on the patient (Phillips et al. 2021), we argue that responsibility can and should be partially shared with the healthcare system. For instance, the hospital can make it easier for the patient to inform her family by providing informational letters, offering genetic counselling sessions for relatives, maintaining resource websites, following up with testing for relatives, and providing specialist referrals if necessary. At a higher level, the government can provide subsidies to reduce the costs of testing and treatments.

We maintain that the socio-relational structures can and *should* mitigate the burden of relational responsibilities. Singapore presents an instructive case study of how this might be achieved. While East Asian cultures generally place care responsibilities for the elderly on the family, ‘what distinguishes Singapore from others is probably that the city-state has implemented an array of policies to regulate and strengthen the role of the family in social support and care provision. It is state familism in action rather than in rhetoric…’ (Zhan and Huang 2023, 113). Singapore utilises three types of policies. First, regulatory policies delineate familial obligations. In addition to the Maintenance of Parents Act, schemes such as ‘Ageing in Place’ and ‘Many Helping Hands’ were set up to cope with an ageing population. These legislations and policies explicitly position the family as the primary caregiving unit (Chia and Lee 2022; Rozario and Rosetti 2012). Second, supportive policies in the form of housing, tax, and financial subsidies provide assistance to the caregivers (Rozario and Rosetti 2012). Third, welfare schemes step in to provide direct state support to the elderly, particularly when family members are unable to do so. These policies include the Pioneer Generation Package (PGP), Silver Support Scheme, and the Silver Support Scheme. What was particularly noteworthy was the shift in Singapore’s stance on welfare with time. In the 1980 s and 1990 s, Singapore had emphasised the family as the first provider of care responsibilities (Teo 2011). More recently however, the state recognised the increasing financial and social challenges on family members and shifted its position to one of shared responsibility between the state and family. The pluralist value of Singapore’s policies — the family has explicit obligations but the burden can be significantly reduced with social resources — is an example of relational responsibility in practice.

In both the familial disclosure and eldercare examples, there are two reasons why responsibilities should be shared with collectives, such as the family and state. First, power and resources enable the system to carry out actions not available to an individual. For instance, Singapore provides assisted living facilities and barrier-free access to help the elderly to ‘age in place’ (Chia and Lee 2022). Second, sharing responsibilities reduces the burden and possible harm on the individual. In the case of filial responsibilities, the rising costs of living in Singapore (Department of Statistics Singapore 2024) meant that family members face increasing challenges in fulfilling these obligations. Further, critics have noted that the burden of care often falls disproportionately on women, causing financial, emotional, and physical stress (Mehta and Thang 2017).

## Relational Responsibility in Practice

Let us return to Sam, whose ageing parent has recently suffered a stroke and now requires long-term, specialised home care. At first glance, Sam appears to face a straightforward moral dilemma: does he fulfil a filial responsibility to care for his parent, or does he prioritise his personal well-being and financial stability? Conventional accounts of special responsibility typically frame this as a dyadic relationship: Is Sam responsible to his parent for the provision of care? A relational responsibility framework, by contrast, opens up a more comprehensive view.

Sam is not only a son; he is also a colleague, friend, and family member. Each relationship generates obligations that compete with his filial responsibilities. For example, if he frequently travels for work or works long shifts, it would be difficult for him to take on caregiving duties. If he is married with children, taking on caregiving responsibilities could lead to burnout. These surrounding relationships matter — not just as external distractions, but as integral elements of Sam’s moral landscape. Furthermore, cultural and legal expectations such as the *Maintenance of Parents Act* in Singapore amplify the pressure on Sam. While this institutional structure is motivated by familial solidarity, it also functions to offload moral and financial responsibility from the state to individuals.

In this case, the healthcare system contributes to Sam’s moral distress by structuring responsibility around individual choice and familial obligation, rather than shared social commitments. The subsidy threshold fails to account for real-life costs and relational constraints, effectively displacing moral responsibility onto family members who are not always willing or able to bear them. Sam’s moral agency becomes constrained not only by internal emotional conflict, but by a socio-institutional environment that treats his filial role as both inevitable and morally binding. Sam’s choices are shaped by laws, policies, and cultural norms that define what is morally expected; even when those expectations conflict with his personal history or current capabilities.

A relational responsibility perspective not only diagnoses these moral pressures, but also reframes ethical possibilities. We can ask: who else is implicated in Sam’s situation? For instance, employers who fail to offer caregiver leave, state agencies that set unreasonably narrow subsidy thresholds, policymakers who uphold rigid legal obligations, and a cultural discourse that equates filial piety with moral worth — these structural actors all participate in creating the conditions for Sam’s moral burden. The ethical response here is not to ask how Sam alone should act, but how society ought to reshape its caregiving infrastructure. This might include developing intermediate subsidy tiers, expanding community-based care resources, supporting flexible work arrangements for caregivers, and promoting public conversations about non-coercive forms of intergenerational support.

In sum, our concept of relational responsibility serves as both a moral lens and a diagnostic tool. It reveals how individual moral distress such as Sam’s is not merely a function of personal failure or moral weakness, but often a symptom of inadequate institutional design and under-recognised relational entanglements. While Sam may still hold some form of responsibility towards his parent, it is only within a fuller accounting of his broader relational and structural context that we can determine what that responsibility realistically and ethically entails. By embedding ethical analysis within the socio-relational environment, we not only gain a clearer understanding of individual dilemmas, but also identify where moral responsibility might more justly be redistributed across collective actors and social systems. This shift opens the way for an ethics of caregiving that is humane, shared, and sustainable.

Future work should develop actionable frameworks for applying relational responsibility in healthcare policy and ethics. This includes exploring how institutions might assess and support overlapping care obligations, mitigate moral distress in family caregivers, and implement fair caregiving infrastructures. Empirical engagement with caregivers’ lived experiences can further inform how socio-relational environments concretely shape moral agency and help refine the practical bounds of relational responsibility.

## Conclusion

Contemporary accounts of special responsibilities often narrow moral focus to dyadic, voluntary relationships between individuals, overlooking the socio-relational environments that give rise to and shape these obligations. In this paper, we have proposed a more expansive and ethically attuned account of relational responsibility that foregrounds the complex web of interpersonal ties, structural conditions, and institutional arrangements in which responsibilities are embedded. Reconceiving responsibilities not individual-centric but polyadic, forming a structured network of obligations allows us to analyse how responsibilities are distributed across multiple agents and contexts. This shift captures the moral complexity of caregiving, where agents are often pulled between overlapping, sometimes conflicting, relationships and social roles. We argued that responsibilities should not be assigned solely on the basis of voluntariness, but on the value of relationships, as determined by those directly involved or affected. Our account thus acknowledges involuntary responsibilities, recognising both the obligations we are born into and the need to protect those to whom responsibilities are owed.

Our account offers a distinct contribution by integrating normative relationality with an explicit structural and institutional analysis. We acknowledge that many responsibilities, particularly those related to care, are involuntary and shaped by historical and systemic forces. Still, such responsibilities must be bounded by moral criteria that protect individuals from unreasonable burdens while securing justice for those in need of care. Finally, we call for a shared ethic of responsibility, in which collectives with the capacity and means should help implement practical solutions to make it easier for individuals to respond to the demands of relational responsibilities. This shared ethic demands not only personal moral responsiveness but structural reforms that redistribute the costs and conditions of care more equitably.

## Data Availability

This is a conceptual paper and does not involve the collection or analysis of empirical data.

## Footnotes

1 Unfortunately, collective responsibility can also result in a diffusion of responsibility, i.e. the situation whereby an individual feels less inclined to take responsibility when other people are present because the individual assumes others can take up the task. Also known in emergency situations as the ‘bystander effect’ (Chekroun and Brauer 2002), there is a wide body of research into this social phenomenon (Beyer et al. 2017), including ways to reduce this problem (e.g. Christensen 2019). Peter French’s (1984) extensive research has shed light on the mechanisms and rationales behind the functioning of collectives.
